# Supplementary material for: The AtMINPP Gene, Encoding a Multiple Inositol Polyphosphate Phosphatase, Coordinates a Novel Crosstalk between Phytic Acid Metabolism and Ethylene Signal Transduction in Leaf Senescence
Source: Int J Mol Sci. 2024 Aug 17;25(16):8969. doi: 10.3390/ijms25168969 (PMC11354338; doi:10.3390/ijms25168969)
Supplement: Supplementary file 1 [file ijms-25-08969-s001.zip › ijms-3142053-supplementary.pdf]

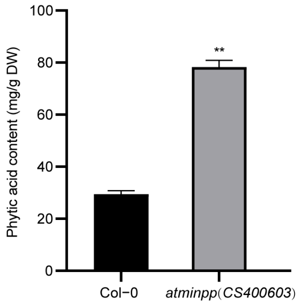

Supplemental Figure S1. Determination of phytic acid content between Col−0 and *atminpp*. Error bars represent the means ± SD (n=3). The significant differences were calculated by Student's *t*−test (***P* < 0.01).

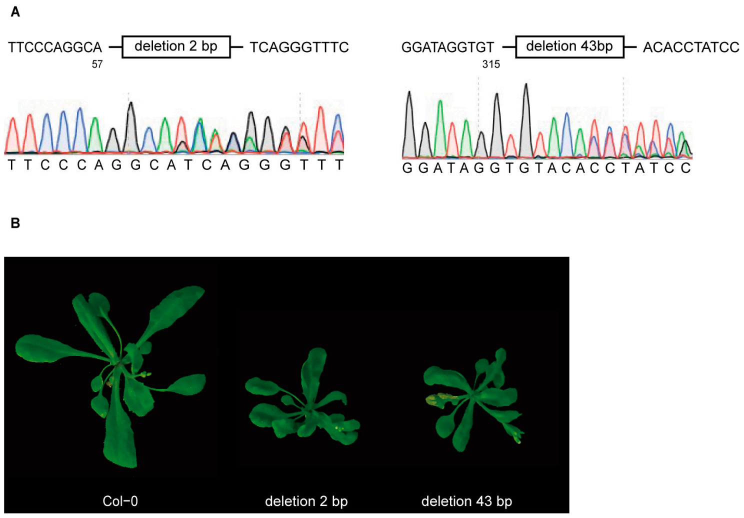

Supplemental Figure S2. Identification and phenotype observation of heterozygous mutants. (A) The representative sequences of two mutant alleles of *AtMINPP* were obtained by CRISPR/Cas9−based genome editing techniques. DNA sequencing peaks showed evidence of successful gene editing in the target region of *AtMINPP*. More peaks represent more mutant alleles. (B) Phenotypes of T_3 transgenic plants of heterozygous *AtMINPP*.

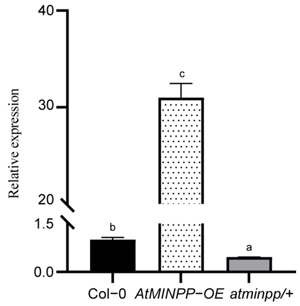

Supplemental Figure S3. Real−time quantitative PCR (RT−qPCR) analysis of transcript levels of Col−0, *AtMINPP−OE* and *atminpp/+*. Error bars represent the means ± SD (n=3). Different lowercase letters above the columns indicate the significant differences according to one−way ANOVA ($P < 0.01$).

Supplementary table:

Table S1. Primers used in this study.

**Primer Name**	**Sequence (5′−3′)**
**RT−qPCR**	
AtMINPP−Real−F	TGGGACCAGGCCGTAATAGA
AtMINPP−Real−R	TCCACAGCAGGCTCTTTAGC
SAG12−Real−F	CGGATGTTGTTGGGCGTTTT
SAG12−Real−R	CCTTCGCAGCCAAAATCGTT
SAG13−Real−F	GGGCTTGGGAGAGAACTCAA
SAG13−Real−R	TGGCTAGTTCCTCCACCAC
SAG29−Real−F	ATCAACTCCTTTGGCTGCGT
SAG29−Real−R	GAGACTTGGAGGGGAGGAGT
SAG113−Real−F	CGGATTTCACTATTGCGGCG
SAG113−Real−R	CCCAGTCAGCATCAGCTTCA
SAG201−Real−F	GGCCAACGTCTCAAGCAAAG
SAG201−Real−R	CAGTCGCCGTCTTTTTGACC
SEN1−Real−F	ACTGATCTTCTCACTGCTGGC
SEN1−Real−R	ACTCTTCTACCGGCAGCTCA
SEN4−Real−F	CGGATTTCACTATTGCGGCG
SEN4−Real−R	CCCAGTCAGCATCAGCTTCA
BFN1−Real−F	TACATCGACACTCCCGACCA
BFN1−Real−R	ATGCTGAAGCTGAGACGTGAA

Primer Name	Sequence (5′–3′)
SIRK–Real–F	AGGAATCGTGGATCAGCGTC
SIRK–Real–R	TGACTCATCGTTGGCCTCTG
ANAC047–Real–F	AGAATATTCGATGAGACTGGATGA
ANAC047–Real–R	TCTGGCGTTTAAGGGGTTGA
TUB2–Real–F	CTCAAGAGGTTCTCAGCAGTACC
TUB2–Real–R	TTTGTGCTCATCTTGCCACGGAAC
**Heterozygous Mutants Screening**	
atminpp/+ LP	GGTGTGCAGATACTCTACTTCG
atminpp/+ RP	CTTGGTCGGAGAACGAGTTC
**ChIP RT–qPCR**	
Locus1–ChIP–F	GTCCCTTTGTATTGTCGGTCTT
Locus1–ChIP–R	GGTTTCTTGATCTTTGCCGGA
Locus2–ChIP–F	CTGGACCTTGAAACTCTTCCATG
Locus2–ChIP–R	GTTTGCACGTTAAACAATCATGAG
Locus3–ChIP–F	GGTCATGCTTTTGACTCAACG
Locus3–ChIP–R	GCCGCCAAATATGCTCTTTG
Negative Control–ChIP–F	GATGCAGAAGCTAGGAAATTGC
Negative Control–ChIP–R	GAACCCGAATTCCCAGTTGGTA
**Vector Construction**	
AtMINPP–OE–F(*Pst* I)	TATCTAGAAAGCTTCTGCAGATGGCGACGAAGACTGTTTGG
AtMINPP–OE–R(*Kpn* I)	TCTTTGTAGTCCATGGTACCCTAGAGCTCGGTATCGTGGCT
proAtMINPP GUS–F(*Pst* I)	TATCTAGAAAGCTTCTGCAGATGGCGACGAAGACTGTTTGG
proAtMINPP GUS–R(*BamH* I)	GACCACCCGGGGATCCCTCTCACGCTTTCTAAATTCCTTCAGGAG
AtMINPP GFP–F(*EcoR* I)	GGCAGCGGCCGAATTCATGGCGACGAAGACTGTTTGG
AtMINPP GFP–R(*Hind* III)	CGGTGGATCCAAGCTTCTAGAGCTCGGTATCGTGGCT
AtMINPP MBP–F(*Nde I*)	ATTTTCAGGGCCATATGATGGCGACGAAGACTGTTTGGATC
AtMINPP MBP–R(*Nde I*)	GCTCGAATTCGGATCCGAGCTCGGTATCGTGGCTTGACCCA
proAtMINPP Y1H–F(*Hind III*)	ATGAATTGAAAAGCTTGTCCCTTTGTATTGTCGGTC
proAtMINPP Y1H–R(*Xho* I)	GAGCACATGCCTCGAGCTCTCACGCTTTCTAAATTCCT
EIN3 Y1H–F(*Nde I*)	CAGATTACGCTCATATGATGATGTTTAATGAGATGGGAATGT
EIN3 Y1H–R(*BamH* I)	CGAGCTCGATGGATCCTTAGAACCATATGGATACATCTTGCT
